# The insecticidal capacity of ethanol extract from *Cascabela peruviana* (L.) Lippold against fruit fly

**DOI:** 10.1016/j.heliyon.2022.e09313

**Published:** 2022-04-21

**Authors:** Tran Thanh Men, Huynh Hong Phien, Tran Thi Tu Ai, Nguyen Van Ay, Nguyen Thi Kim Hue, Do Tan Khang, Tran Duy Binh

**Affiliations:** a Department of Biology, College of Natural Sciences, Can Tho University, Cantho City 900000, Viet Nam; b Department of Plant Physiology and Biochemistry, College of Agriculture, Can Tho University, Cantho City, 94000, Viet Nam; c Department of Molecular Biotechnology, Biotechnology Research and Development Institute, Can Tho University, Cantho City, 94000, Viet Nam; d Department of Functional Chemistry, Kyoto Institute of Technology, Kyoto 606-8585, Japan

**Keywords:** *Cascabela peruviana* (L.) Lippold, Flavonoid, Insecticidal activities, Polyphenols, Toxicity

## Abstract

*Cascabela peruviana* (L.) Lippold (*C. peruviana*) has been extensively used for its antifungal and antibacterial properties. However, its role in anti-insect is still under investigation. To investigate the ability of the ethanol extract of *C. peruviana* against insects, we used the fruit fly (*Drosophila melanogaster*) as a model to gain more insight into the toxic effects of this extract. We found that the ethanol extract from the stem and leaves of *C. peruviana* was effective against insects and contained polyphenol and flavonoid compounds. *C. peruviana* could induce mortality of 2^nd^-instar larvae and reduce growth and reproduction of fruit flies. Interestingly, the toxicity of *C. peruviana* extract has been remained to affect the development of the next generation of fruit flies. The locomotor activity and feeding ability of the F1 generation of this insect were significantly reduced by *C. peruviana*. In addition, flavonoids and polyphenols, as well as saponins and tannins were detected in the ethanol extract of *C. peruviana*. We assume that the ability of the extract of *C. peruviana* to control insects may be related to the presence of high levels of these compounds. The findings highlighted that the extract from the leaves of *Cascabela peruviana* has the potential to be used as an insecticide.

## 1 Introduction

Vietnam is located in a tropical monsoon area with hot and humid climate favorable for the development of insect pests. These insects not only cause significant damage to various plants, but are also vectors for animal and human diseases. Our country is the third-largest rice exporter in the world after India and Thailand. In 2017, rice yield decreased by up to 30%, caused by insect pests. They can even destroy the entire crop (Donatelli et al., 2017). In addition, the damage caused by insects also reduces the quality of agricultural products, leading to a drop in prices. Most importantly, many insects are intermediate hosts for the transmission of serious diseases to humans and animals, such as dengue fever and malaria. These diseases are not only a problem for tropical countries, but also pose a major problem for global public health. As a result, various methods of pest control are used in the form of insecticide-resistant crops. Insecticides are most commonly used in practice. However, they are dangerous to the polluted environment, humans, consumers, beneficial predators, and parasitoids. Therefore, it is urgent to explore substances or compounds with insecticidal activity from natural sources.

Fruit flies are considered serious pests, and the cost of infestation has been estimated to be millions of dollars annually worldwide (Abraham et al., 2015). *Drosophila melanogaster* is known as a fruit fly because it is interested in the smell of fermented fruit. The genus *Drosophila* includes many species of fruit flies that can cause damage. For example, *D. mojavensis* and *D. erecta* use cacti and screw pines, respectively, as food sources. However, instead of damaging plants by ingestion, *Scaptomyza flava* parasitizes on leaves of the Brassicaceae family by laying its eggs and producing offspring. In addition, *Drosophila* has also attacked other plants such as the fruits of *Ficus capensis*, mushrooms, and even bananas (Anholt, 2020). In Vietnam, fruit flies, *Bactrocera dorsalis*, *B. correcta*, cause fruit rot on dragon fruit, cashew nuts, mangoes (Dieu and Huynh, 2009). Maize was infested by *Spodoptera frugiperda*, resulting in lower yields and higher pesticide and labor costs (Nguyen and Gilleski, 2020). In addition, the brown planthopper (BPH), *Nilaparvata lugens*, is a species of planthopper that destroys *Oryza sativa* L. by feeding directly on and transmitting two viruses, rice ragged stunt, and rice grassy stunt virus (Khush, 1999). BPH is the most important rice pest and the main factor reducing rice yields in Vietnam (Lamba and Dono, 2021).

*Drosophila* has become a powerful insect model system to answer questions in insect physiology and toxicology (Scott and Buchon, 2019). Because of its genetic and physiological similarities to mosquitoes and other insects, the use of *Drosophila* as an insect model system has been proposed to provide an excellent understanding in the field of insect toxicology, including attempts to aid in the screening of effective insecticides (Schneider, 2000). Furthermore, *Drosophila* has advantageous characteristics, including a short life cycle, good reproductive ability, and ease of husbandry. In addition to these properties, it is also compatible with many types of pests, and a highly sensitive experimental system has been established to test the insecticidal activity of plant extracts (Savadogo et al., 2021). Moreover, established libraries of *Drosophila* mutants and transgenes, as well as transgenic and reporter lines, are available, suggesting that *Drosophila* offers the opportunity to pursue innovative experimental approaches that are thought to be difficult to implement in other insect model systems (Pandey and Nichols, 2011). Consequently, *Drosophila* may replace the other insects as an insect model for studying the insecticidal effects of plant extracts.

*Cascabela peruviana* (L.) Lippold (CP) or *Thevetia peruviana* (Pers.) K. Schum, native to Mexico and Central America, is used as a medicinal plant to heal ulcers, scabies, and hemorrhoids and dissolve tumors. *Cascabela peruviana* (L.) Lippold seed oil has been shown to be resistant to termites and the European corn borer (*Ostrinia nubilalis*) (McLaughlin et al., 1980; Obasi and Igboechi, 1991). The aim of this study was to find natural substances capable of controlling insect pests that are environmentally friendly, effective and sustainable in an agricultural system. The data showed that the ethanolic leaf extract of *C. peruviana* significantly inhibited fruit fly development. Unlike other extracts, the toxicity of *C. peruviana* leaf extract could be sustained to kill the next generation of fruit flies by preventing locomotor activity and feeding. All in all, we suggest that *C. peruviana* leaf extract may be promising for use as an insect control agent.

## 2 Materials and methods

### 2.1 Preparation of CP ethanol extracts

The stems and leaves of *Cascabela peruviana* (L.) Lippold were collected in Can Tho City, Vietnam, naturally dried, and ground with a stick blender. 10 g of the dried stems or leaves from CP were extracted with 100 mL of ethanol (FUJIFILM-Wako, Wuxi, China) at room temperature for 24 h, then filtered through filter paper (Whatman No. 1) to collect the supernatants. The residues were extracted three more times and the supernatants were combined. The CP extracts (CP-stem and CP-leaf) were concentrated under pressure in a rotary evaporator (Rotavapor R-300, BUCHI, Switzerland), freeze-dried, and then stored at 4 ^○^C until use. Plants were identified by Dr. Nguyen Thi Kim Hue and kept in the Laboratory of Plant Biology, Department of Biology, Can Tho University, Vietnam, under code numbers (CPst09.2019-CT006 and CPl09.2019-CT006).

### 2.2 Phytochemical analysis

One gram of crude extract of stem and leaves of CP was dissolved in 100 mL of water to obtain a stock of concentration 1% (*v/v*). The crude extracts obtained were determined for their chemical constituents such as alkaloids, flavonoids, phenolic compounds, saponins, and tannins by qualitative methods of natural compounds described below.

Detection of alkaloids: Add 1 mL of crude extract to the test tube and treat with a few drops of Mayer’s reagent. Alkaloids are precipitated and formatted with white-yellowish turbidity by Mayer’s reagent, which is a neutral or slightly acidic solution (Warsi and Sholichah, 2017).

Detection of saponins: Add 5 mg of dried extract to the test tube and shake vigorously with 5 mL of distilled water to obtain a stable, persistent foam. If the foam persists for 15 min, it indicates the presence of saponins (Kareru et al., 2007).

Detection of flavonoids: A few drops of sodium hydroxide solution were added to 1 mL of the crude extract. Flavonoids react with sodium hydroxide solution to generate acetophenon (yellow color), which is colorless when diluted with acid (Warsi and Sholichah, 2017).

Detection of polyphenolics: Three drops of ferric chloride solution were added to 1 mL of the crude extracts. Phenols form a complex with ferric ions, which have bluish-black color (Warsi and Sholichah, 2017).

Detection of tannins: One mL of 1% gelatin solution and 1 mL of 10% sodium chloride was added to 3 mL of the crude extracts. Tannins cause the precipitation of gelatin from the solution (Shah and Hossain, 2014).

### 2.3 Quantitative determination of total polyphenol, flavonoid content

The total polyphenol content of CP-stem and CP-leaf was determined using the modified Folin–Ciocalteu’s assay (Singleton et al., 1999). First, the reaction mixture consisting of 250 μL of extract, 250 μL of water, and 250 μL of Follin-Ciocalteu’s reagent was shaken well. Then, 250 μL of sodium carbonate solution (Na_2_CO_3_; 10%; *w/v*) was added to the solution. The sample was mixed by vortex and incubated at 40 °C for 30 min. The absorbance at 756 nm was measured using a SH-1200 microplate reader. Gallic acid was employed as a positive control to make the standard curve equation. The total polyphenol content of each extract was calculated using the gallic acid standard curve equation. The results were expressed in milligrams of gallic acid equivalent (GAE) per gram weight of dried extract (mg GAE/g extract).

The total flavonoid content in CP-stem and CP-leaf was analyzed by the aluminum chloride (AlCl_3_) colorimetric method with a minor modification (Shi et al., 2018). The reaction mixture containing 200 μL of the extract or standard at the concentration studied and 200 μL of distilled water was reacted with 40 μL of sodium nitrite (NaNO_2_; 5%; *w/v*). The reaction mixture was mixed well and then left for 5 min. After 5 min, added 40 μL of the aluminum chloride (AlCl_3_; 10%; *w/v*) to the mixture and mixed well. The reaction was mixed and incubated at room temperature for 6 min before adding 400 μL of sodium hydroxide 1M (NaOH) and distilled water to reach a volume of 1 mL. Absorbance at 510 nm was measured using a SH-1200 microplate reader. Quercetin was used as a positive control. Total flavonoid content was determined as mg/g quercetin equivalent (QE) of the dry extract.

### 2.4 HPLC analysis

The dried CP leaf extract (3 g) was heated at 70 °C for 30 min (to hydrolyze glycosides to aglycons) in a solution of 50 ml of 70% aqueous methanol in which 0.5 g/L tert-butyl hydroquinone (TBHQ) was dissolved, and 10 mL of 6 N HCl was added. After cooling, the solution was sonicated for 5 min and made to a final volume of 100 mL by adding deionized water. The solution was then filtered through a 0.2 μm anotop syringe filter for HPLC analysis. An HPLC system with Pum L2130 and an UV-VIS L-2420 detector (Hitachi, Japan) was used. Samples were loaded onto a Cosmosil 5C18 MS-II column (150 mm × 4.6 mm, Nacalai Tesque, Inc., Japan). Compounds were eluted using a gradient elution of mobile phases A and B. Solvent A consisted of deionized water and 0.1% formic acid and solvent B consisted of methanol and 0.1% formic acid. Flavonoids were detected at 280 nm at a flow rate of 1 mL/min.

Rutin and quercetin standard solutions were prepared and injected until their peaks were seen on an HPLC chromatogram. Each standard solution (0.1–0.5 μg/mL rutin and 0.1–2.0 μg/mL quercetin) was dissolved in methanol. Calibration curves were plotted based on the average peak areas against the concentration of each analyte. The flavonoids were identified by matching the retention time and their spectral characteristics with those of the standards, and the amount of flavonoids was calculated from the calibration curves.

### 2.5 Feeding assay

The 2^nd^-instar larvae were fed a standard diet containing CP extracts (0 mg/mL and 200 mg/mL) and 2.5% (w/v) brilliant blue R for 24 h. Ten larvae were homogenized with 200 μL of ddH_2_O and then 800 μL of ddH_2_O were added. The supernatant was collected with filter paper. Absorbance was measured at 630 nm using a SH-1200 microplate reader.

### 2.6 The influence of CP extracts on the mortality of the 2^nd^-instar larvae of fruit fly

The *wild-type* strain used was Canton-S (CS). This fly was gifted from Prof. Kaeko Kamei (Kyoto Institute of Technology, Japan). Fly strains were reared at 25 °C on standard food (4% dry yeast, 9% cornmeal, 10% glucose, 0.8% agar, 0.5% propionic acid, and 0.05% ethyl parahydroxybenzoate) (Men et al., 2016). Thirty male and ten female (1:3) CS flies were mated and kept at 25 °C for 1 day. They were then transferred to a new tube containing standard food for 2 h for oviposition to obtain a synchronized larval age (Duy Binh et al., 2019). During desired larval growth, 2^nd^-instar larvae were collected for the assays.

The influence of extracts from CP on the mortality of 2^nd^-instar larvae was conducted using the modified method described previously (Riaz et al., 2018). CP-stem and CP-leaf extracts (1 g/mL) were completely dissolved in distilled water, which was used as the stock solution. The stock solution was prepared using the appropriate sample concentration before use. The standard food was supplied with a range of CP-stem or CP-leaf concentrations (0, 30, 60, 90, 120 and 150 mg/mL). The insecticide pertox was used as a positive control (Marcus and Fiumera, 2016). Forty 2^nd^-instar larvae were selected and kept in each food vial. Each treatment was repeated 5 times (5 vials). The percentage of dead larvae after 7 days and the total number of eclosed flies after 10 days were monitored to evaluate the effect of the extracts of CP on the mortality of 2^nd^-instar larvae.

### 2.7 The influence of CP extracts on the growth of fruit flies

Thirty male and ten virgin female flies of the strain CS were mated on standard food and kept at 25 °C for 1 day. They were then transferred to a new tube containing CP extracts (20 mg/mL) and standard food for 24 h for oviposition. Various developmental indices of 3^rd^-instar larvae, pupae and adult flies were analyzed, such as number, body weight, pupation, eclosion of pupae. Moreover, the appearance of abnormal phenotypes was used as an index to evaluate the effect of CP extracts on fruit fly growth (Chowański et al., 2018). The adult flies obtained in the experiment were referred to as P generation.

### 2.8 The influence of PC extracts on the fertility of fruit flies

The obtained male and virgin female flies of P generation were mated on the standard food for 24 h. The number of pupations and eclosion of the adult flies were measured to determine the effect of the extracts of CP on the fecundity of fruit flies (Ferdenache et al., 2019). The adult flies obtained in this experiment were referred to as the F1 generation.

### 2.9 Climbing assays

Fly climbing experiments were performed as previously described (Valéria Soares de Araújo Pinho et al., 2014). 14-day-old male flies from the F1 generation were anesthetized with CO2 and placed in empty plastic test vessels marked at a height of 6 cm from the bottom of the test vessel. After 30 min, the flies fully regained consciousness and acclimated to the conditions in the test vessels. Then, the vials of the treatments were tapped simultaneously to ensure that the flies in the vials fell completely to the bottom. The number of flies that climbed above the predetermined 6-cm mark was recorded within 10 s. Each treatment was repeated five times.

### 2.10 Statistical analysis

The experiments were repeated at least three times. Excel 2016 and GraphPad Prism version 9.2.0 (332) were used for statistical analysis and figure preparation. Data are presented as mean ± standard deviation. Statistical significance of differences was determined using an unpaired *t* test, a two-tailed *t* test, or a one-way analysis of variance. *P* < 0.05 was considered statistically significant.

## 3 Results and discussion

### 3.1 Qualitative phytochemical screening of CP extract

Preliminary phytochemical analysis of the stem and leaf extract of CP revealed phenolic compounds, flavonoids, and tannins. Alkaloid compounds were absent in both leaf and stem, while saponins were present only in the leaf extract of CP (Table 1). Secondary defense compounds could be inactively stored or produced in response to insect or bacterial attack (War et al., 2012). The bioactive compounds, such as phenolic compounds, alkaloids, benzoxazinoides, cyanogenic glucosides, glucosinolates, and terpenoids, effectively controlled insect pests (Fürstenberg-Hägg et al., 2013).

**Table 1:** Qualitative results of natural compounds present in the extracts of *C. peruviana* stem and leaf.

Extract	Alkaloid	Flavonoid	Tannin	Saponin	Phenolic
Stem	-	+	+	-	+
Leaf	-	+	+	+	+

Note: (+): positive; (-): negative.

A previous study indicated that the insect resistance ability of many weed species depends on the presence of chemical components such as flavonoids, saponins, tannins, steroids, cardiac glycosides, alkaloids, anthraquinones, and terpenoids (Riaz et al., 2018). In line with the above results, Attaullah et al. (2020) demonstrated that the extracts from different weeds were potentially effective against the development of *Musca domestica* fly (Diptera, Muscidae). These extracts contained many flavonoids, steroids, saponins, cardiac glycosides, tannins, alkaloids, terpenoids, and anthraquinones (Attaullah et al., 2020). Thus, the extract of *C. peruviana* contain compounds that are potentially resistant to insects.

### 3.2 Quantitative analysis of CP extract

**Figure 1:**
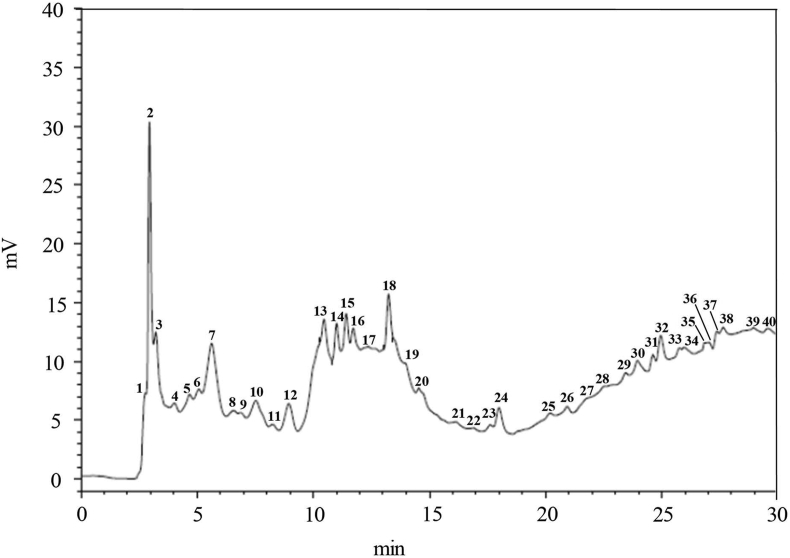
HPLC chromatogram of CP leaf extract analyzed at 280 nm and identified with retention time (min) Peak No. 13 (rutin) and 18 (quercetin) were identified as peaks.

The data showed that the extracts of CP contained high levels of polyphenols and flavonoids. The total amount of polyphenols and flavonoids in the leaf extract was 238.6 ± 3.68 mg GAE/g dry extract and 60.5 ± 3.00 mg QE/g dry extract, respectively, higher than that of the stem extract (Table 2). Therefore, the CP leaf extract was analyzed by HPLC. As shown in Figure 1, the HPLC chromatogram of the extract displayed 40 peaks detected at 280 nm. Two flavonoid compounds (rutin and quercetin) were identified from the leaf extract by matching their retention times with those of the standards. Peaks 13 and 18 were identified as rutin and quercetin, respectively, with good resolution. In addition, the concentration of these flavonoid compounds was quantified. The research results showed that rutin was 1.29 mg/g of dry extract and quercetin was 0.84 mg/g of dry extract.

**Table 2:** Polyphenols and flavonoid compounds contained in the extract of *C. peruviana* stem and leaf.

Extract	Polyphenols (mg GAE/g dried extract)	Flavonoid compounds (mg QE/g dried extract)
Stem	200.10 ± 2.21	12.13 ± 1.43
Leaf	238.56 ± 3.68	60.51 ± 3.00

Furthermore, a study showed that extracts of purple maize (*Zea mays*) suppressed the growth of *Manduca sexta* eggs (Tayal et al., 2020), suggesting that purple maize hulls have the potential to inhibit *M. sexta* development. Interestingly, the total polyphenol content of the purple corn husk extract (186.6 ± 1.03 g GAE/g) was lower than the polyphenol content of the CP extracts, indicating that the CP extract may inhibit insect growth through its polyphenols and flavonoid compounds. The ability of flavonoids to prevent insects was demonstrated by evaluating their feeding behavior, growth, and fecundity (Onkokesung et al., 2014). These suggest that the plant extract, which contains polyphenols and flavonoid compounds, deters insects.

### 3.3 Effect of CP extracts on mortality of fruit fly larvae

**Figure 2:**
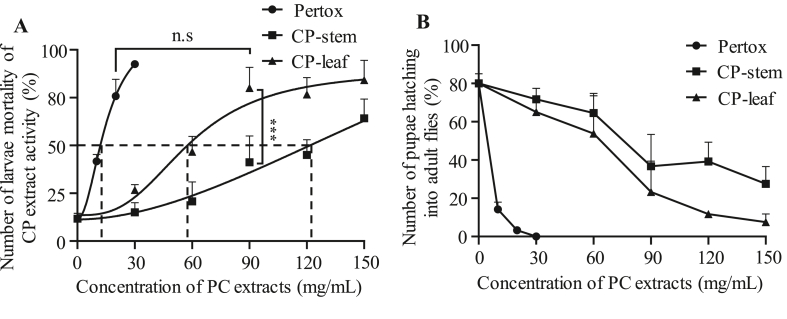
The effect of CP extract on fruit fly mortality. 2^nd^-instar larvae were fed standard food and different concentrations of CP stem and leaf extract. Pertox was used as a positive control. The number of pupations was calculated after 7 days to confirm the mortality of fruit fly larvae (A), while the eclosion of flies was evaluated after 10 days (B). The dotted line indicates the concentration of stem and leaf extract of PC or the control substance required for 50% killing the 2^nd^-instar larvae (LD_50_). Data are presented as means ± SD. Statistical significance was calculated by *t* test. ∗∗∗, *p* < 0.001; n. s, not significant.

To test whether 2^nd^-instar larvae consumed the diet containing the extracts of PC, feeding ability was indirectly assessed by the absorption of brilliant blue R using a feeding assay. These data showed that the intake of the standard diet containing CP extracts had no significant difference from the standard diet without CP extract, indicating that CP extract was initially insensitive to the fruit fly larvae (data not shown). Subsequently, the efficacy of CP extract against insects was investigated. The 2^nd^-larval stage of fruit fly was cultured for 10 days at 25 °C on a standard diet containing a serial concentration of the stem and leaf extract of CP (0, 30, 90, 120 and 150 mg/mL), and then the number of eclosed flies was counted. Besides, the mortality of larvae after 7 days was calculated based on pupation. The results indicated that the extract of CP stem and CP leaves significantly caused the death of 2^nd^-instar larvae and suppressed the eclosion of fruit fly in a dose-dependent manner (Figure 2). Remarkably, at a concentration of 90 mg/mL, CP-leaf extract could induce larvae mortality by 80% at 7 days and suppress the eclosion by 76.7% at 10 days compared with controls (0 mg/mL). The lethal dose (LD_50_) of leaf and stem extract required to kill 50% of 2nd-instar larvae of the fruit fly was 59.1 mg/mL and 122.5 mg/mL (equivalent to 15.3 mg/mL of Pretox), respectively (Figure 2A). As shown in Figure 2, the ability of CP-leaf extract to induce fruit fly larval mortality was up to 1.8 times stronger than that of CP-stem extract at the same concentration. These results were consistent with the results of the quantitative analysis in Table 2. These data support the conclusion that plant extracts containing polyphenols and flavonoid compounds have the potential to repel insects. To clarify the insect repellent properties of extracts from CP, we used pertox, a commercial insecticide, as a positive control at different concentrations of 0, 10, 20, and 30 mg/mL. Compared with the positive control at 20 mg/mL, the CP-leaf extract at a concentration of 90 mg/mL showed no significant difference to induce fruit fly larval death. Moreover, the insecticidal activity of the extract of CP was better than other plant extracts to inhibit the growth of fruit flies (Attaullah et al., 2020; Riaz et al., 2018). These results suggest that the CP-leaf extract may contain harmful compounds for insect growth.

Many studies suggest that polyphenols and flavonoid compounds extracted from plants benefit our health at appropriate concentrations (Nguyen et al., 2020a, b). However, these compounds also serve as a defense mechanism against insect attacks. It is well documented that the insect gut plays an essential role in growth physiology through feeding, digestion, and nutrient uptake (Franzetti et al., 2015). Therefore, pest control has emerged as an effective strategy by inhibiting food metabolism in the insect digestive system. A previous study indicated that azadirachtin, the main component of the neem plan (*Azadirachta indica*), not only inhibited α-amylase activity in midgut epithelial cells, but also disrupted the structure of the larval epithelium of *Plodia interpunctella*, thereby disrupting nutrient digestion (Rharrabe et al., 2008). It was concluded that the extracts of CP could suppress the development of fruit fly larvae by a similar mechanism.

### 3.4 Effect of CP extracts on the development of fruit fly

**Figure 3:**
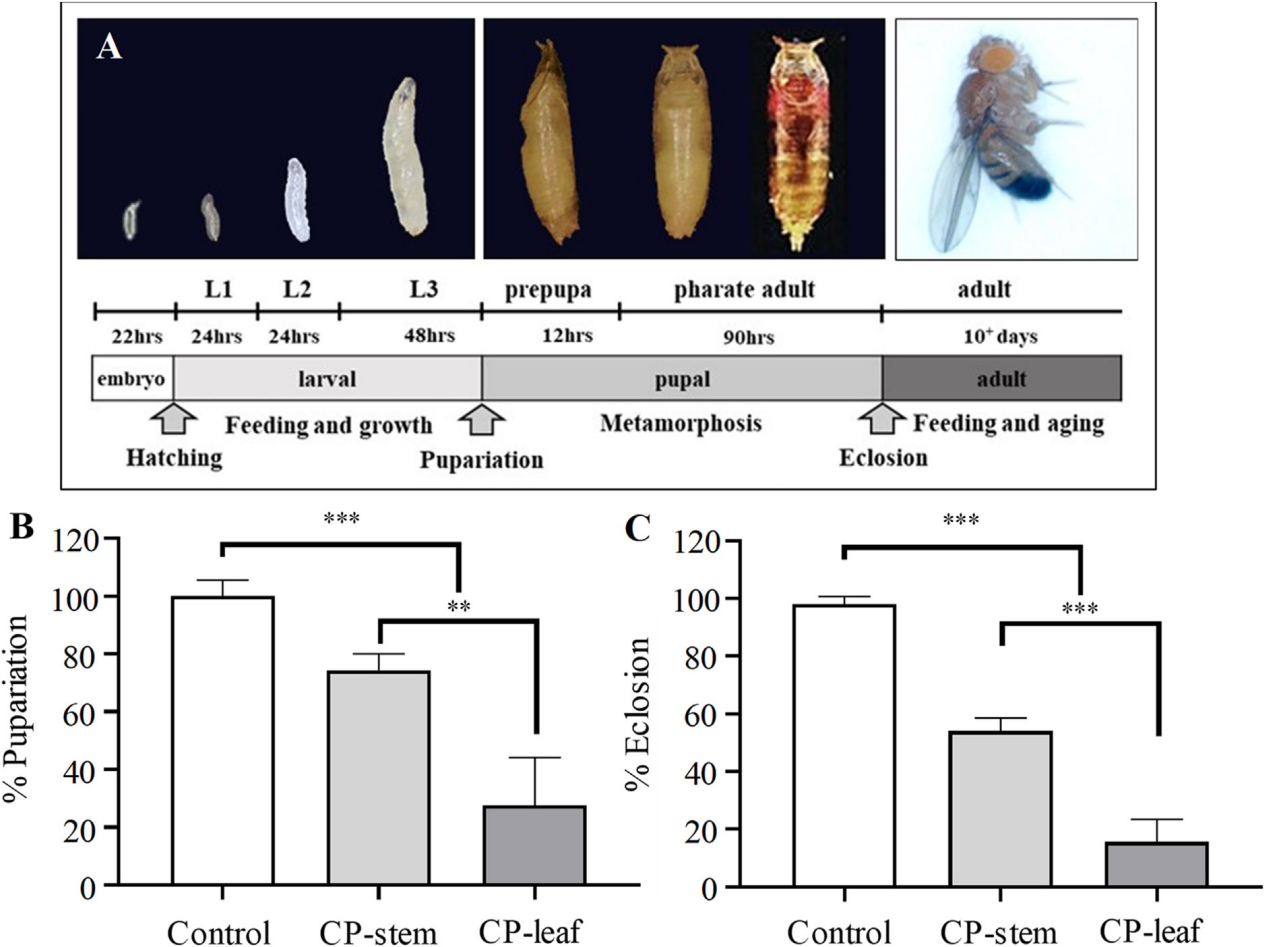
The extracts of PC suppress the development of fruit flies. Fruit fly development from embryo to adult is holometabolous and proceeds through the intermediate stages of larva and pupa. The approximate developmental times for each stage at 25 °C and the transitional events that can be assessed (e.g., hatching, pupation, and eclosion) are indicated (A). The percentage of pupation (B) and eclosion of flies (C) treated with or without PC extracts was analyzed (*n* = 3, 100 pupae or flies for each treatment). L1, L2, and L3 denote the 1^st^, 2^nd^, and 3^rd^ larval stages of the fruit fly, respectively. Statistical significance was calculated by *t* test and one-way ANOVA. ∗∗, *p* < 0.01; ∗∗∗, *p* < 0.001.

Thirty male and ten virgin female flies of the strain CS were allowed to lay eggs on standard food or on standard food containing CP extracts (20 mg/mL), and then the inhibition of fruit fly pupation and eclosion was examined (Figure 3A). As expected, the extracts of CP strongly suppressed these two processes. Moreover, the results showed that larvae fed with the stem and leaf extracts significantly decreased pupation (1.4-fold and 3.6-fold, respectively) than the control (0 mg/mL) (Figure 3B). Moreover, fly eclosion was not only drastically suppressed (Figure 3C), but delayed by 2 days by the CP extracts at this concentration. A previous study has shown that azadirachtin can affect insect development by prolonging the life of larval or pupal stages and inhibiting the molting process (Vinuela et al., 2000). The results suggest that the delayed lifespan of fruit flies caused by CP extracts may be related to the presence of azadirachtin in these extracts. Another possibility is that polyphenols and flavonoid compounds, as secondary metabolites, affect the hormone ecdysone and thus interfere with cytochrome-P450, which regulates the molting process in insects (Sztal et al., 2012).

**Figure 4:**
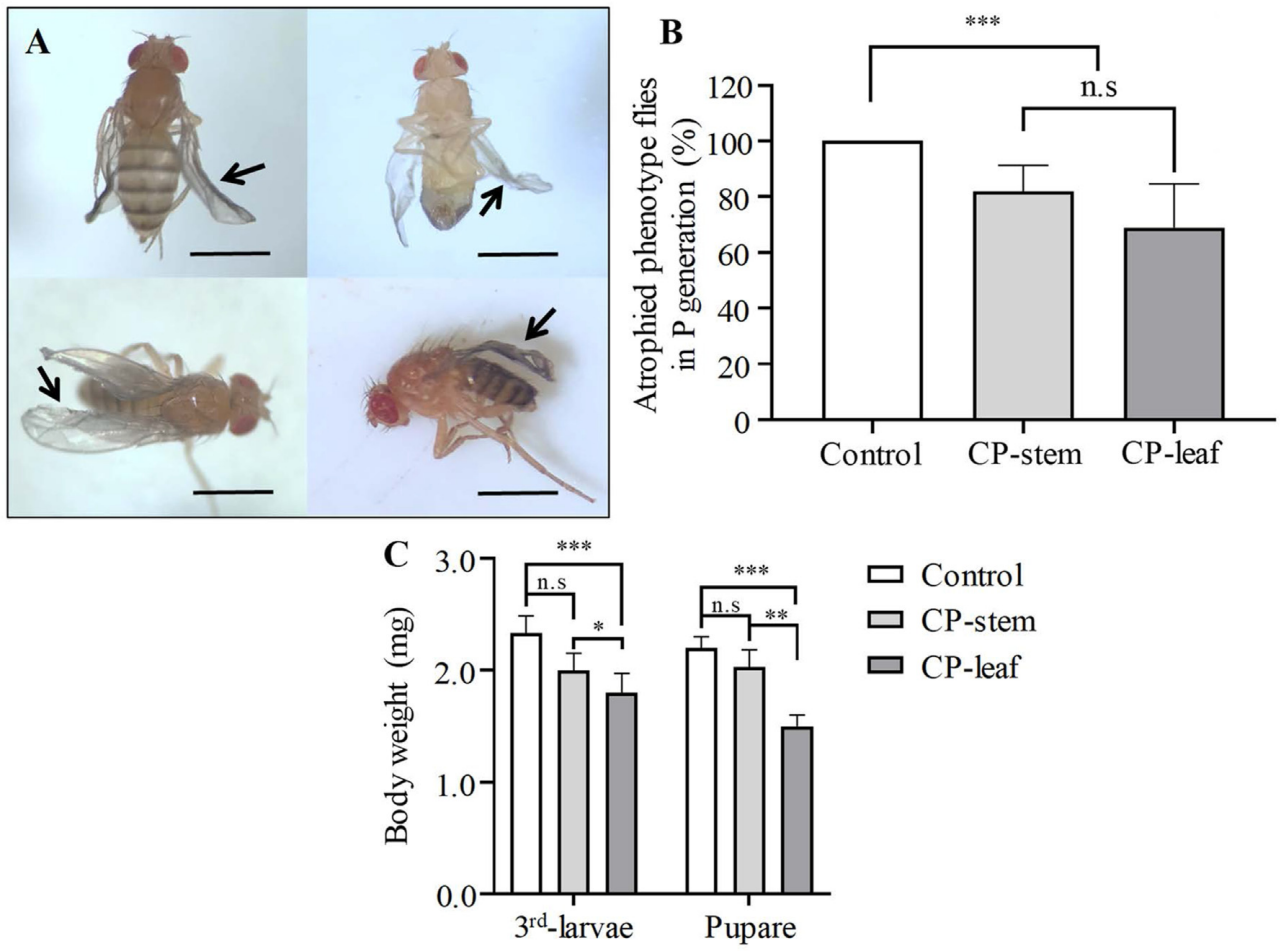
Extract of PC reduces body weight of 3^rd^-instar larvae and pupae and results in abnormal phenotypes in adult fruit flies. Shown are microscopic images of adult flies. Fruit flies showed abnormal phenotypes (A). The percentage of fruit flies with atrophic phenotypes is shown in (B) (*n* = 3, 100 flies per replicate). Body weights of 3^rd^-instar larvae and pupae treated with or without PC extracts were analyzed (*n* = 3, 100 larvae or pupae per replicate). The black arrows indicate the abnormal phenotypes. Statistical significance was calculated by *t* test and one-way ANOVA. Scale bar, 1.0 mm. n.s, not significant; ∗∗, *p* < 0.01; ∗∗∗, *p* < 0.001.

Several studies have demonstrated that the abnormal morphology of the fruit fly caused by consumption of plant extracts is small body size, stunted wings, and abdomen. In addition, other phenotypes were also noted, especially on the wings of fruit flies, such as sticking together and reduced locomotion. To further evaluate the effect of the extracts of PC on fruit fly development, the phenotypes of adult flies, generation P, were observed. As shown in Figure 4, the atrophied phenotypes occurred (approximately 18.1% of the stem extract and 31.0% of the leaf extract). These results are consistent with the effect of *S. tuberosum* and *L. esculentum* leaf extracts on the growth and reproduction of *Drosophila* (Ventrella et al., 2016). Remarkably, the percentage of abnormal phenotypes in these extracts was lower than in the leaf and stem extracts from CP.

Moreover, body weight is also an indicator of fruit fly development. Only leaf extract from CP affected the weight of 3^rd^-instar fruit fly larvae and pupae (Figure 4C). After 7 days of feeding with standard food containing the leaf extract of CP, the weight of the larvae was reduced by 1.8 times compared to the control. Not surprisingly, pupal weight also decreased by 1.5-fold. As explained above, plant extracts could inhibit the digestion and absorption of food during larval development, causing weight reduction. Lack of nutrients is one of the reasons why the larvae could not pupate and the mortality of fruit flies increased (Vinuela et al., 2000). Corio et al. (2013) reported that the reduction in larval weight was closely related to food intake. In this study, it was found that the body weight of *D. buzzatii* larvae fed with this extract was reduced by the effect of *T. terscheckii* extract on the metabolic process of this fly (Corio et al., 2013). These data supported the above findings that the ability of CP extracts to inhibit fruit fly development may be related to food digestion.

**Figure 5:**
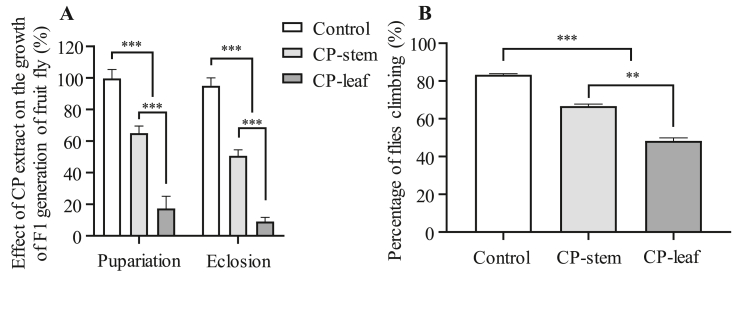
Intake of CP extract suppresses F1 generation development and reduces movement activity of fruit flies. The percentage of pupation and eclosion of flies treated with or without PC extracts was analyzed (A) (*n* = 3, 100 flies per replicate). Fruit fly locomotion in the F1 generation was reduced by feeding with PC extracts (*n* = 5, 20 flies per replicate). Statistical significance was calculated by *t*-test and one-way ANOVA. Scale bar, ∗∗, *p* < 0.01; ∗∗∗, *p* < 0.001.

To determine whether the pupation and eclosion of fruit flies in the next generation continue to be affected by these extracts, the ability of fruit flies to reproduce was examined by assessing the development of the F1 generation. The results showed that the F1 generation of fruit flies was affected in their pupation and eclosion because their parents were previously fed with CP extracts (Figure 5). Moreover, the leaf extract had the strongest effect, reducing pupation by 3.8-fold compared to the stem extract and 5.8-fold compared to the control (0 mg/mL). We then examined the number of flies hatched from the pupae to determine the effects of the plant extracts on the eclosion stage. Our results showed that the number of adult flies decreased 0.2-fold (stem extract) and 0.5-fold (leaf extract) compared to the control (Figure 5A). de Souza et al. (2021) found that the essential oils of *B. calvescens*, *B. mesoneura*, and *B. oblongifolia* can reduce the fecundity of *D. suzukii* flies (de Souza et al., 2021). Lucantoni et al. (2006) reported that azadirachtin affects the fertility of mosquitoes (*Anopheles stephensi*) by inducing the abnormal ovarian structure, preventing oogenesis and spermatogenesis, and impairing the formation of the vitelline shell of this species (Lucantoni et al., 2006). In addition, azadirachtin has been shown to reduce the ability to successfully mate in *D. melanogaster* and to affect the number and size of follicles and oocytes (Oulhaci et al., 2018).

### 3.5 Intake of CP extract reduces the locomotion of fruit flies

Climbing ability is one of the indicators that reflect the health condition of fruit flies. To obtain more evidence of the insecticidal potential of the extracts of CP, the locomotion of the adult fruit fly was analyzed by a climbing test. Therefore, the aim of the experiment was to analyze the locomotion of adult flies of F1 generation treated with extracts of PC at the age of 14 days. The influence of the plant extract was determined by the number of flies that were able to climb above and below the 6-cm mark on the test tubes. Figure 5B shows that the extract from the leaf and stem of CP effectively inhibited the movement ability of fruit flies. A previous study demonstrated that the hydroalcoholic extract of *D. furfuracea* leaves can completely inhibit fruit fly locomotion at a concentration of 50 mg/mL (Valéria Soares de Araújo Pinho et al., 2014). Another study indicated that the inhibition of fruit fly locomotion by the extract of *D. furfuracea* leaves was related to the parameters of locomotion behavior, acetylcholinesterase (AChE) activity, and the enzyme involved in the release of the neurotransmitter acetylcholine in the insect central nervous system (Kim and Lee, 2013). These results suggest that the effects of the extract of CP on the climbing ability of fruit flies may be related to these factors.

## 4 Conclusions

The extracts from the leaf and stem of *Cascabela peruviana* contain flavonoids, polyphenols, alkaloids, saponins, and tannins. The results of quantitative screening showed that the extract from the leaves of *C. peruviana* had higher content of polyphenols and flavonoids than the extract from the stem. The extracts from the leaves and stems of *C. peruviana* were effectively toxic to the 2^nd^-instar larvae by affecting the fecundity and development of fruit flies, and especially by restricting the locomotion of fruit flies. Further studies are needed to confirm that the insecticidal effect is related to nervous system enzymes such as AchE. Our results provide the first evidence that *C. peruviana* leaf extract suppresses insect development and maintains its efficacy from the first to the second generation. Therefore, further research is needed to isolate the active compounds and evaluate their effects on insects.

## Declarations

### Author contribution statement

Tran Thanh Men; Tran Duy Binh: Conceived and designed the experiments; Analyzed and interpreted the data; Contributed reagents, materials, analysis tools or data; Wrote the paper.

Huynh Hong Phien: Performed the experiments; Wrote the paper.

Tran Thi Tu Ai; Nguyen Thi Kim Hue: Performed the experiments.

Nguyen Van Ay; Do Tan Khang: Contributed reagents, materials, analysis tools or data.

### Funding statement

This research did not receive any specific grant from funding agencies in the public, commercial, or not-for-profit sectors.

### Data availability statement

The authors do not have permission to share data.

### Declaration of interests statement

The authors declare no conflict of interest.

### Additional information

No additional information is available for this paper.
